# Automatic detection and extraction of key resources from tables in biomedical papers

**DOI:** 10.1186/s13040-025-00438-9

**Published:** 2025-03-20

**Authors:** Ibrahim Burak Ozyurt, Anita Bandrowski

**Affiliations:** FDI Lab Dept of Neuroscience, UCSD, 9500 Gilman Drive M/C 0608, La Jolla, CA 92093-0608 USA

**Keywords:** Table extraction, Scientific reproducibility, Information extraction, Natural language processing, Language modeling, Bioinformatics

## Abstract

**Background:**

Tables are useful information artifacts that allow easy detection of missing data and have been deployed by several publishers to improve the amount of information present for key resources and reagents such as antibodies, cell lines, and other tools that constitute the inputs to a study. STAR*Methods key resource tables have increased the “findability” of these key resources, improving transparency of the paper by warning authors (before publication) about any problems, such as key resources that cannot be uniquely identified or those that are known to be problematic, but they have not been commonly available outside of the Cell Press journal family. We believe that processing preprints and adding these ’resource table candidates’ automatically will improve the availability of structured and linked information about research resources in a broader swath of the scientific literature. However, if the authors have already added a key resource table, that table must be detected, and each entity must be correctly identified and faithfully restructured into a standard format.

**Methods:**

We introduce four end-to-end table extraction pipelines to extract and faithfully reconstruct key resource tables from biomedical papers in PDF format. The pipelines employ machine learning approaches for key resource table page identification, “Table Transformer” models for table detection, and table structure recognition. We also introduce a character-level generative pre-trained transformer (GPT) language model for scientific tables pre-trained on over 11 million scientific tables. We fine-tuned our table-specific language model with synthetic training data generated with a novel approach to alleviate row over-segmentation significantly improving key resource extraction performance.

**Results:**

The extraction of key resource tables in PDF files by the popular GROBID tool resulted in a Grid Table Similarity (GriTS) score of 0.12. All of our pipelines have outperformed GROBID by a large margin. Our best pipeline with table-specific language model-based row merger achieved a GriTS score of 0.90.

**Conclusions:**

Our pipelines allow the detection and extraction of key resources from tables with much higher accuracy, enabling the deployment of automated research resource extraction tools on BioRxiv to help authors correct unidentifiable key resources detected in their articles and improve the reproducibility of their findings. The code, table-specific language model, annotated training and evaluation data are publicly available.

## Background

A table is defined (by the Oxford English dictionary) as an arrangement of numbers, words, or items of any kind (content aspect), in a definite and compact form (structure aspect), so as to exhibit some set of facts or relations in a distinct and comprehensive way, for convenience of study, reference, or calculation (function aspect).

Resource tables in scientific papers, such as the STAR*Methods key resources table [1] denote the use of chemical reagents, antibodies, cell lines, organisms, software tools, instruments and other experimental input to the study. Many of these types of research resources are associated with erroneous reporting practices [2], and have been called out by the community as the main culprits and sources of variability that lead to the reproducibility crisis [3]. The STAR*Methods key resource table became a very powerful tool in biomedicine because these simple three-column data structures provide immediate visual stimulus highlighting missing information that might ordinarily be hidden in long paragraphs, encouraging the author to verify names and look up identifiers for resources. Before these tables were implemented, the percentage of antibodies that were identifiable hovered between 10 and 30%, preventing direct replication of 70 to 90% of antibody-using articles, but after the implementation of the STAR*Methods key resource tables in the journal Cell, more than 95% of antibodies were identifiable [4]. The ability to determine which reagent is used in a particular study is a major source of irreproducibility in the scientific literature [5], and a source that should be “easy to fix”, as most authors simply need a quick reminder to pull the appropriate information from laboratory records into the paper [6]. The practice of looking up identifiers allows authors to confront bad lab practices such as insufficient record keeping and also enables alerts that an issue may have been reported about a resource used. Information about the quality of research resources may be difficult to find; for example, the use of the “Willoughby-Hoye Python Scripts” has been shown to produce an error when analyzing NMR shifts [7], however while remembering to check whether there are these kinds of issues is difficult, an alert attached to the RRID record for the scripts that clearly showing the warning may help authors identify problems before they stake their reputations on faulty resources (RRID:SCR_017562). Therefore, resource tables that ask authors to look up or verify persistent identifiers such as RRIDs are a highly effective means to improve transparency and fidelity of scientific work.

The ability of STAR*Methods key resource tables to reduce omitted resource information in the scientific literature is directly related to their use. Unfortunately, most journals do not enforce a standard resource table due to the lack of manpower to enforce this type of policy (personal communication). The preprints submitted to BioRxiv, the major preprint server for biology, are not checked by editorial staff except to determine if the manuscript is obviously a scholarly work in biology and thus may be the place where this information omission is most acute, but as preprints are not yet published, they may be a perfect place for intervention. Unlike the Cell Press journals, these tables in BioRxiv are in arbitrary formats and the BioRxiv team creates images of tables to maintain their human readability while reducing their ability to be operated on because the task of coding tables into a more interpretable format is too resource intensive (Richard Sever, personal communication). This task requires that tables created by authors in arbitrary formats be found and correctly interpreted as to the table structure and cell values, which has been beyond the scope of the BioRxiv team. To help authors of BioRxiv preprints, we undertook the task of automatically creating resource tables for BioRxiv preprints based on PDF versions of the documents because the more structured versions contained images of tables. With detection and text processing, we should be able to provide the information to authors as automatically created tables and encourage them to look over the result and fix any errors of omission that were introduced by either the process of writing the manuscript or the extraction of text into tables. However, for authors who already include a resource table in their preprint, we must detect them and display the results with high fidelity.

Wang [8] provided a formal model for the logical structure of a table. A table consists of entities, the basic data that it displays, and labels, the auxiliary data used to locate the entities. The labels are further classified into categories that form a tree structure. Based on this model, a table is a mapping of entries with frontier label sequences from a tree-structured set of categories. During table construction, the logical structure of a table is converted into a two-dimensional grid structure for representation. Table data extraction is the reconstruction of the underlying implicit logical model of a table from its representation. Viewed from the perspective of natural language processing (NLP), this task is made more demanding by two factors. First, there are long-distance relationships between table labels and entities that do not fit human language syntax and semantics. Second, there is almost no redundancy in the entities and labels to learn syntax and semantics. Attacking the problem from the representation side using computer vision techniques is another approach to infer the logical structure from the layout. However, this approach requires optical character recognition (OCR) to convert the content of detected table cells back to text, resulting in character-level errors (even an OCR method with 99% accuracy will have a wrong character every 100 characters on average). To illustrate why even this low an error rate may be problematic we can imagine that we are examining a PDF file of an invoice which must be paid, but one of the numbers is incorrect without any indication as to which one is incorrect leading to potential problems. Nearly any character can be found in a catalog number or an organism name, and even one mistake in 100 will invariably lead to a different item.

**Fig. 1:**
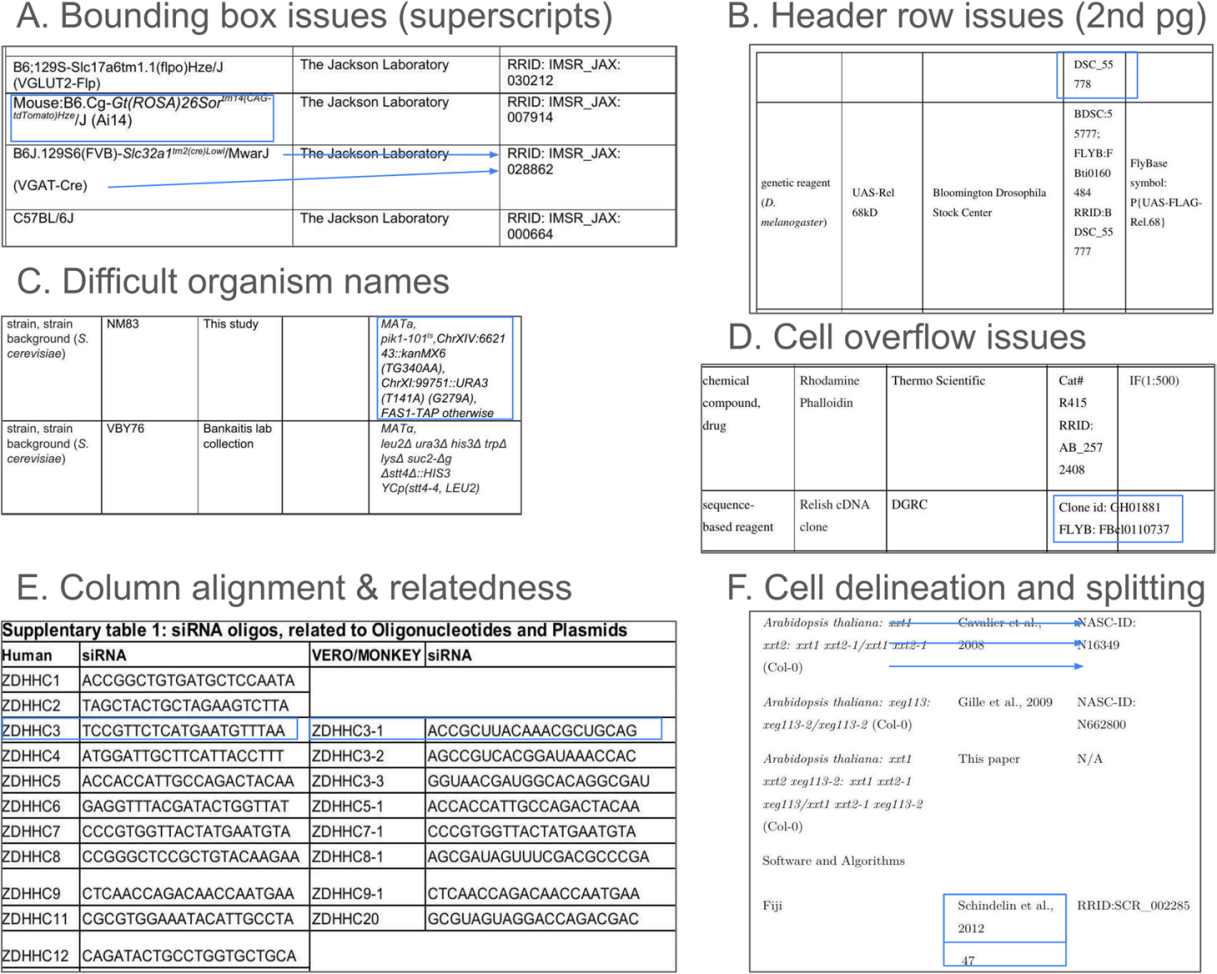
Common issues with key resource tables

Figure 1 summarizes some of the most commonly encountered issues in resource tables, and they include problems such as places where superscripts or other characters are very close to the bounding boxes (A), creating potential errors in OCR because the bounding box may be interpreted as part of the character. Some tables that are present on more than a single page may only have one header row and some cells may be broken between page one and page two causing errors in data stitching, in the case shown in (B) the first row on page 2 of the table contains a part of the identifier that is associated with the fly on the previous page. Some organism names are fairly long and may have multiple lines with characters that are more similar to math symbols than English names (see C). In a relatively common case for BioRxiv preprints, authors sometimes do not notice or fix cases where the contents of a single cell overrun that cell, and in at least one case the overflow is not visible because it was covered by a white box in the next cell and only shows up in the text-extracted version of the table. Tables are also not all structured in the same way, and we cannot assume that a row always means that the information content is about the same entity. In fact, in part (E), the table contains a set of oligonucleotides that are similar between humans and monkeys, but they are not the same reagents. Many tables do not contain any bounding box information, that makes it more difficult to know where one cell begins and another ends. In part (F), the bounding box at the bottom contains a reference and a page number, which might be stitched together, and information in two cells that take three rows in the leftmost cell, two lines in the middle cell, and two lines in the rightmost cell. Knowing that the information should be stitched together, not one row at a time but three rows, two rows, and two rows, respectively, is not trivial for a table extraction system.

### Related Work

While early approaches for table extraction where mostly rule and heuristics based [9, 10], current approaches [11–13] rely on deep learning-based object detection on images of document pages. These approaches typically use convolutional neural networks (CNN) and/or recurrent neural networks (RNN), requiring a large amount of labeled training data.

Preparation of the training data for image-based approaches involves labeling of bounding boxes for each table cell for a large number of tables, which is a very resource-intensive task. PubMed Central provides millions of articles and preprints in NISO Journal Archiving and Interchange (JATS) XML format, also encoding tables occurring in the corresponding papers. In addition to the JATS XML files, the corresponding PDF files are also provided. By associating the tables encoded in JATS XML files with their corresponding PDF character locations, a large set of labeled table data can be generated. Using this approach, training/evaluation sets are generated such as PubTabNet [13] and PubTables-1M [14] for table extraction. Adams et al. [15] introduced a benchmark set of 1650 tables for neurological disorders.

The importance of supplementary materials, such as tables for the extraction of the scientific literature, is demonstrated in genomics [16]. Starting from PMC OAS JATS XML encoded tables of clinical papers, Milosevic et al. [17] developed a framework to extract numerical (patients, age) and textual (adverse effects) information. The information from tables that were detected by table classification was also used to detect anticancer drug-side effect relationships [18]. In scientific and biomedical domains, GROBID [19] is currently the most popular tool for extracting, parsing, and re-structuring of PDFs into structured XML/Text Encoding Initiative (TEI) encoded documents including tables. Recently, the Table Transformer [14] model trained on tables extracted from the PMC OAS (PubTables-1M) was also introduced.

Even after the recent advances in machine learning and artificial intelligence, automated approaches cannot perform at the level of human experts. Given enough resources, human expert annotations can work at a large scale, as shown by the NCBI’s large-scale manual annotation of PubMed articles with standardized MeSH terms. Until the transition to automated indexing in 2022, all PubMed articles were assigned standardized MeSH terms by domain experts [20].

Most of these approaches assume that the tables are represented in a structured format such as JATS XML eliminating the need for table and structure recognition. However, this information is only available in the open-access subset of PMC after an open-access paper has been published. In order to detect key resources that cannot be identified in a paper and warn the authors to correct them, the intervention must be done before publishing, at the preprint step. At this step most preprints are in PDF format. To this end, we introduce four end-to-end key resource table extraction pipelines together with a GPT language model to represent the language of scientific tables, which is fine-tuned with a novel synthetic training data generation scheme for learning to merge over-segmented table rows.

## Methods

### Systems Overview

**Fig. 2:**
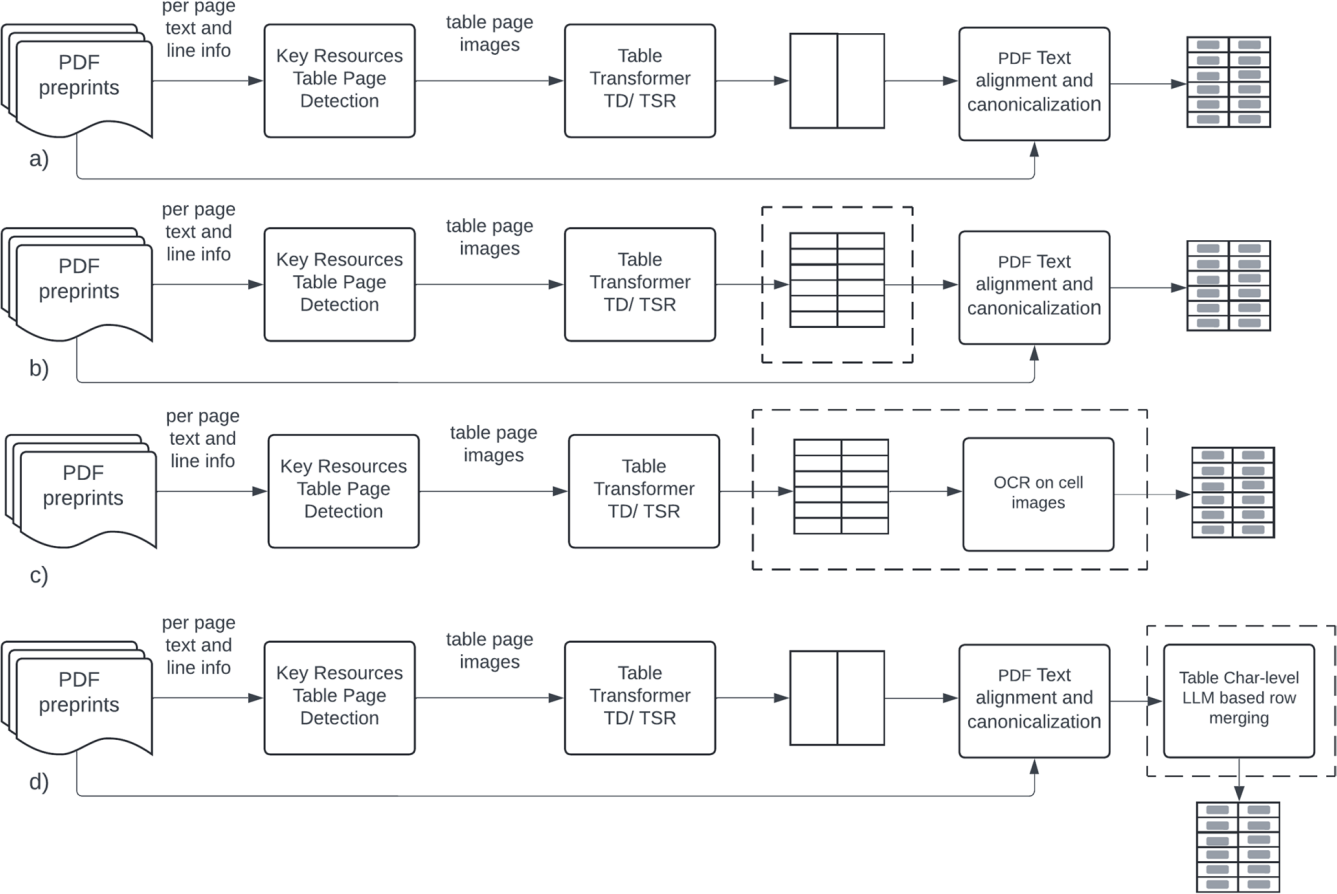
Key resource table reconstruction pipelines. **a** RTE-Col (using only TSR column location info) **b** RTE-Row (using both TSR column and row location info) **c** RTE-OCR (fully image based using OCR for cell contents) **d** RTE-LM (using only TSR column information together with character level table language model based row merging). The differences in pipelines are highlighted by enclosing dashed boxes

Four pipelines are introduced to automatically detect key resources from tables found in PDF preprints and supplementary documents, as summarized in Fig 2. The key resource candidate detection module is common to all four pipelines. It selects the candidate pages in a paper to be used in the following pipeline module which uses the Table Transformer table detection (TD) model to detect table bounding boxes, followed by cell bounding box prediction via the Table Transformer table structure detection (TSR) model. The resource table extraction (RTE) pipelines RTE-Col, RTE-Row, and RTE-LM are hybrid, multi-modal approaches relying on image-level models to detect table, column, and (for RTE-Row) row boundary information followed by the extraction of the corresponding cell text from the PDF file. Extraction of the text from the PDF for each cell identified by the TSR bounding box information (whether it is just column range or both column and row range information) is done by aligning the character bounding boxes and applying canonicalization steps on the candidate strings which are detailed in following sections. Column ranges derived from the TSR model’s predicted cell boundary boxes were observed to be less error-prone than row ranges. The separation between table columns is almost always larger than the separation between table rows in table images. Based on this observation, RTE-Col only uses column ranges derived from TSR model cell boundary box predictions, resulting in over-segmentation of the table rows with overflowing cells. RTE-Row pipeline uses both column and row ranges, derived similarly from the TSR model predictions. To remedy the row over-segmentation problem resulting in spurious rows for overflowing cells, RTE-LM uses a language model-based approach to merge over-segmented table rows reliably after the RTE-Col pipeline. RTE-OCR pipeline works on the image modality alone, where the text from the TSR predicted bounding-box images is extracted using optical character recognition.

### Detection of Resource Table containing Pages in PDFs

**Fig. 3:**
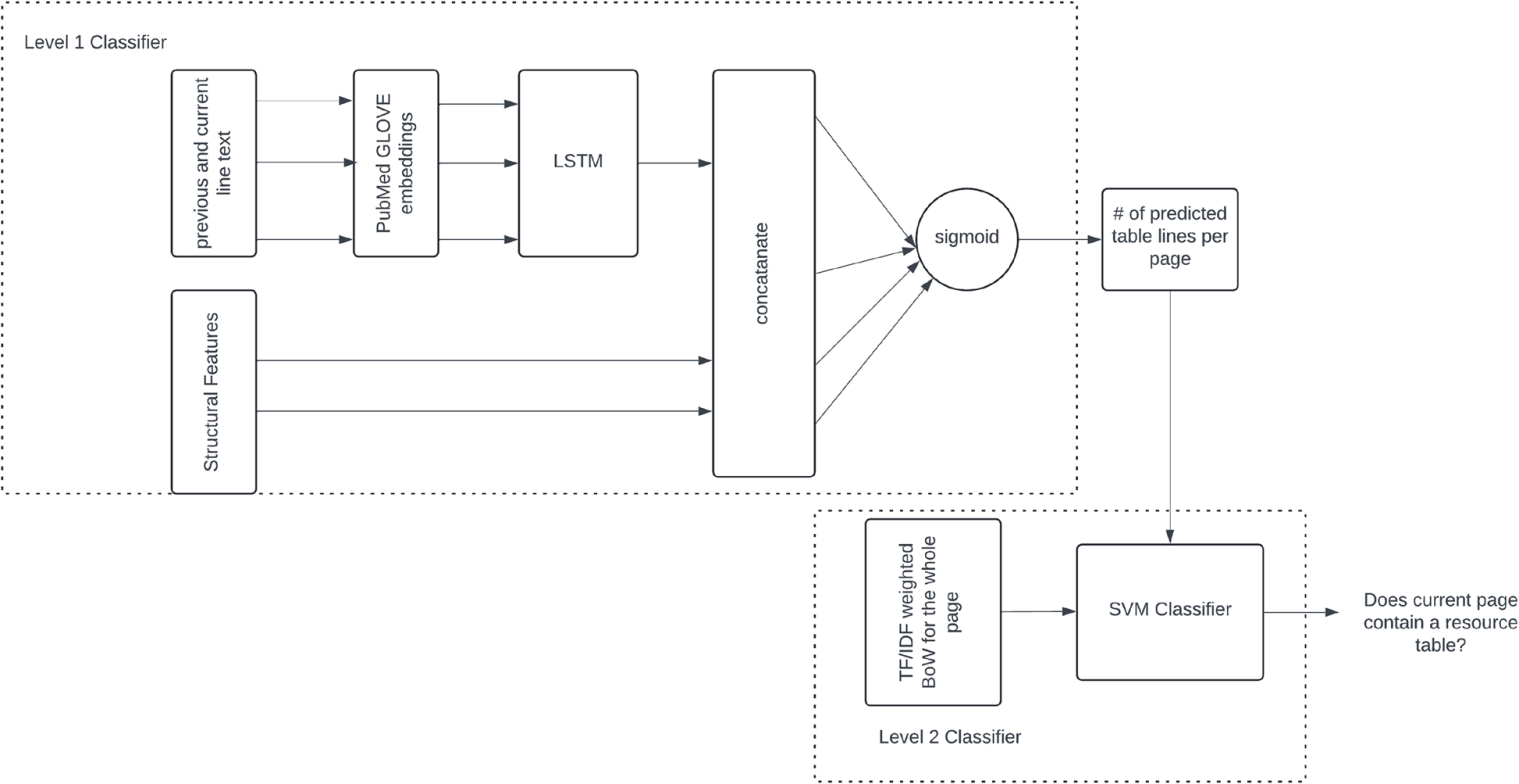
A stacked generalizer for detection of key resource table containing pages in a PDF preprint

Since we are interested in the detection of a specific type of table, namely the key resources table, PDF pages that contain key resources tables need to be detected before the detection of tables. After table detection, their structure and finally reconstruction of their content can commence. In PDFs, each character has its own bounding box [$x_{min}$, $y_{min}$, $x_{max}$, $y_{max}$] and shapes such as grid lines of a table are represented as polygons with specified vertex coordinates. Since not all tables have a grid and other document structures such as figures are also constructed from primitive polygons, by themselves grids are not indicative of a table occurring in a table when they are detected. Identifying pages that potentially contain key resource tables is approached using a two-level stacked generalizer [21] ensemble classifier where the predictions of the first-level classifier are used as additional features for a second-level classifier making the final decision. The overall classifier ensemble architecture introduced is illustrated in Fig 3.

The first-level classifier operates on local line-level features to predict whether the current line of a PDF document page is inside a key resource table or not. For this classifier, a long-short-term memory (LSTM) [22] based neural network is employed using both text-based and structural features. Using a windowing approach, the tokens of the current line and any previous line constitute the local textual context. For structural features, any vertical and horizontal lines that are encoded in PDF primitive graphics operations for the current and any previous line are used as indicator features. The current page number is also used as a feature. As token embeddings, GloVE [23] word vectors trained in-house on PubMed abstracts were used. In the neural network, the textual context embedded as pre-computed GloVE word embeddings is encoded using LSTM neurons and concatenated via the other structural indicator features for a final logistic regression layer. To compensate for the imbalance in the number of lines of text in a table versus not, the errors in the positive class are penalized 50 times more than the negative class errors during training.

The second level classifier is a linear support vector machine (SVM) operating on the text content of the whole page together with the number of in table lines predicted by the first level classifier. By using a very common text classification assumption, bag of words (BoW) assumption, the unique words in the training corpus, are represented by their TF-IDF score to model their relevance in the current page. Since the number of pages that are not key resource tables is at least an order of magnitude more than key resource containing tables, to compensate for the imbalance in negative and positive classes, errors in the positive class are penalized five times more than the negative class in the objective function for the SVM classifier.

To train and test the introduced system, 57 BioRxiv preprints containing key resource table(s) were manually labeled by extracting their text content, including page numbers, and marking the beginning and the end of each resource table or pages for multiple page-spanning tables. The labeled preprints were randomly split into a training set of 47 preprints and a testing set of 10 preprints. After preprocessing, 69,632 training and 9,152 testing instances are generated for the first-level LSTM classifier.

### Table Detection and Table Structure Detection using Table Transformers

Table Transformer [14] models were trained on 948K tables from PMC OAS corpus (RRID:SCR_004166). JATS XML table cell contents are aligned with the corresponding PDF text boxes via the Needleman-Wunsch algorithm [24]. The authors also introduced a canonicalization algorithm to correct over-segmentation errors in the structure annotation of a table (i.e., merging adjacent cells under certain conditions) [14]. The authors fine-tuned a CNN-Transformer object detection model, DETR [25], with a ResNet-18 backbone trained on Imagenet data for table detection (TD) and Table Structure Detection (TSR). For this work, we have used Table Transformer models available from Hugging Face (RRID:SCR_020958), https://huggingface.co/docs/transformers/main/en/model_doc/table-transformer).

### Object Detection to PDF Alignment and Canonicalization

To facilitate the alignment of the Table Transformer TD and TSR bounding boxes with the corresponding PDF document text bounding boxes, first, the bounding box coordinates are scaled to PDF page coordinates as the Table Transformer model preprocessing involves image scaling. This is followed by column and row range estimation and heuristic row merging steps described in the following sections.

#### Column and Row Range Estimation

The effective number of columns of a table is predicted as the median number of cells in a TSR detected row. After that, for each column, the lower bound is the minimum $x_{min}$ value and the upper bound is the maximum $x_{max}$ value of all detected cell boundary boxes for that column plus about 1 pixel additional space to minimize column boundary alignment errors. The bound of the last column from the Table Transformer TSR bounds is expanded by two average character widths to compensate for occasional clipping of last characters due to tight bounding-box errors.

For pipelines without row information from Table Transformer TSR model, row ranges are estimated by first grouping all character position bounding boxes in the PDF document by their top *y* coordinate followed by merging all characters that are connected horizontally resulting in text lines. Since superscript and subscript character bounding boxes have different top *y* values than their baseline, this operation creates spurious text lines which are handled by a separate canonicalization step.

#### Canonicalization (Row Merge Phase)

Using only the more robust table column range information estimated based on the Table Transformer TSR model results in over-segmentation of the table rows. Besides that, any superscripts/subscripts are raised/lowered from the baseline text in PDF extracted text lines. To decrease the number of spurious rows generated, following heuristic rules are used;

1. If a row text range overlaps 70% or more with the closest row rectangle range, merge both rows (mostly for subscripts and superscripts)
2. If a row has empty cells and a row above without empty cells and row text range overlaps 50% or more with the closest row text range, merge both rows

While these rules work in many situations, they cannot handle all cases.

### Optical Character Recognition for Table Cells

For the RTE-OCR pipeline, we extracted the table cell content images based on the TSR model predicted bounding boxes. For OCR, we used the open-source Tesseract [26] software. Each cell image was first converted to gray scale followed by contrast enhancement before Tesseract OCR processing. We used the ’Assume a single uniform block of text’ option of Tesseract to minimize OCR errors.

### Learning the Language of Scientific Tables

Human language generation is traditionally modeled as a joint probability distribution of a sequence of conditional probabilities of tokens denoted as a language model [27]. Based on this model, given a sequence of symbols $(s_1,s_2,\dots ,s_n)$ such as words or characters in a sentence, the conditional probability of generating the next symbol $s_k$ depends on the previous context expressed as $p(s_k|s_1,s_2,\dots ,s_{k-1})$. Current advances in deep learning architectures, specifically Transformer architecture [28], allowed increasingly more expressive models of these conditional probabilities by increasing the width and height (number of transformer layers) of the underlying neural network.

For training corpus preparation, the PMC OAS June 2024 set of full papers in JATS XML format is used. From these papers, 11,467,759 tables are extracted by parsing their corresponding JATS XML file. From each table, the content of each cell, including the header rows, is used to build our pre-training corpus of about 1.7 billion tokens.

Since a table cell usually contains condensed information articulated with a few words or numbers and with the ultimate goal of being able to predict if the content in the potentially over-segmented next row under the current cell is the continuation of the current cell, a language model at character level instead of the most common word piece level is used. Thus, an auto-regressive generative language model is trained to predict the next character given the characters up to the next character for all the cell contents of all tables available in PMC OAS papers.

A generative pre-trained transformer (GPT) [29] architecture with decoder transformer layers using causal multi-head self-attention is selected as the language model. Causal self-attention, unlike the transformer encoder models such as BERT [30], only attends to the tokens before the predicted next token. A six-transformer layer GPT model with six attention heads and 384-dimensional embedding vectors is used to model the language of the table contents of biomedical papers. The resulting model has 15.6 million parameters and can handle sequences up to 256 characters. A vocabulary of 11,946 unique characters and ‘<EOS>’ special token to indicate the end of the sequence (i.e., the end of table cell content) is used. The language model is pre-trained on an RTX 4090 24 GB GPU with a batch size of 256 for 250,000 steps in 8 hours.

### Learning to merge overflowing table cell content

A solution to the table row over-segmentation problem for the extracted table data reconstruction is learning to classify if the contents of two vertically neighboring cells should be merged or not. Being able to do this requires domain knowledge such as recognizing different representations of organisms, cell lines, antibodies or genetic sequences, plasmids and ability to exploit syntactic and semantic level language clues such as a hyperlink spread across multiple rows. Our hypothesis is that a character-level language model pre-trained on the contents of a large number of table cells will implicitly learn the syntactic and semantic properties of the language used in biomedical domain tables to represent results and data characteristics. Given a table of *C* columns and *R* rows, where a cell content of the ith row and jth column is denoted as $c_{ij}$, the binary classification problem for a single table can be stated as $f: \textbf{X} \rightarrow y = \{0,1\}$ where $\textbf{X} \in \{c_{ij}|<\! EOS \!>| c_{i+1,j}, 0<= i< R-1, 0<= j < C\}$. Here, *y* is a binary label indicating whether $c_{ij}$ and $c_{i+1,j}$ should be merged or not, and | is the concatenation operator. For transfer learning, the head layer of the character level table LM is replaced with a single sigmoid neuron, and the rest of the model is initialized from the weights of the character level table LM.

### Generating simulated table cell overflow dataset

To train the supervised cell merge classification, a large labeled training set of key resource-containing tables is necessary which is costly and time-consuming to annotate. However, cell merge-requiring scenarios can be simulated from the contents of JATS XML represented tables in PMC OAS. To do this, first, the key resource containing tables from more than 11 million tables in the PMC OAS dataset need to be selected reliably. Since RRIDs are used increasingly with key resource tables, we filtered PMC OAS tables for the ’RRID:’ prefix resulting in 13,664 tables to create a simulated cell merge training set.

To simulate one or more cells overflowing into multiple rows, all tables having at least one row over the size of 90 characters (the maximum number of characters to fit a single line on a page with a reasonable font size) are selected. For tables with less than 100 max character total width, defined as the length of all its column contents of its longest row, an 80-character width maximum is used. The allowed total row width is divided to each column in proportion to their average column width plus the standard deviation of the column’s width. This process allocates additional space for columns with a greater variation in widths and simulates the way the authors select column widths to minimize column spillovers. After the column widths are determined, any cells that do not fit their allocated column width will be candidates for positively labeled cell merge pairs. Negative training examples can be generated from non-overflowing cells in neighboring rows of the same column. A simulated overflowing cell is split at space characters if possible to as many rows as necessary to represent the cell content.

### Row Merge Prediction

The 13,664 key resource table corpus is randomly divided into a 90%/10% training/testing set. Using this approach, 2.8 million simulated training instances and 311,998 testing instances are generated. The binary classification model is trained for two epochs using the Adam optimizer and a learning rate of 2e-4. The model achieved an accuracy of 98.7% on the test set of key resource tables.

Since the introduced key table resource cell merger classifier only predicts whether two rows in consecutive cells of the same column should be merged, a maximum voting based approach is used to decide which neighboring table rows to merge. To achieve this for any pair of table rows, the cell merger classifier was applied to each column cell pair and a row score was generated by summing the predicted column cell merge probabilities and finding the average value. If the row score is over 0.5 then the neighboring rows were merged.

### Key Resource Table Extraction Gold Standard Set Construction

Evaluating of the introduced approaches requires a gold standard set of reconstructed tables to test against. Due to the lack of such a resource, the April 2024 collection of 4652 BioRxiv preprints were downloaded. Preprints containing the keywords ’RRID’ and ’antibod’, common keywords potentially indicative of key resources, were selected resulting in a set of 1655 candidate preprints, which are processed by the RTE-Col pipeline resulting in 302 preprints with key resource tables detected. Of this set, 50 preprints were randomly selected for manual correction of over-segmentation errors to generate the gold standard set. The final gold standard set contains 100 tables in 46 BioRxiv preprints and was used to evaluate the key resource table extraction pipelines introduced.

### GROBID Baseline Table Extraction

As a baseline, GROBID [19] system is also tested against our constructed gold standard table set. The corresponding PDF documents of the gold standard tables were processed via the latest GROBID server (version 0.8.0) installed locally, followed by the parsing of the recognized tables from the generated XML/TEI documents. After that, the GROBID recognized tables were aligned with the gold standard set by the vocabulary overlap of the GROBID table with the corresponding gold standard table. An overlap in vocabulary over 40% is considered as a threshold for table alignment to maximize the inclusion of partially extracted tables by GROBID.

### Evaluation Metrics

To evaluate both the topology and content of the tables extracted from biomedical paper/supplementary document PDFs, Grid Table Similarity (GriTS) [31] measure is used. GriTS represents the ground truth and predicted tables as matrices and computes the two-dimensional most similar substructures among these matrices as shown in Eq. 1 taking fully into account the two-dimensional structure of tables and addressing the cell topology, location, and content in a unified manner. Here, $\tilde{\textbf{A}}$ and $\tilde{\textbf{B}}$ denote the substructures, a selection of rows and columns aligned between two table matrices $\textbf{A}$ (ground truth) and $\textbf{B}$ (predicted). Three different similarity functions *f* are defined for cell topology, content, and layout similarity between ground truth and predicted table. Cell content and topology similarities are the most relevant for the extraction of key resource tables.

$\mathrm {GriTS_{Cont}}$ measures the similarity of the layout and content of cells, while $\mathrm {GriTS_{Top}}$ measures the similarity of the row and columns that each cell occupies on the grid between the ground truth and the predicted table. GriTS measure can be interpreted as the commonly used *F* measure, which is the geometric mean of recall and precision. For GriTS, the precision and recall are defined in Eqs. 2 and 3, respectively. The similarity of cell contents between the aligned ground-truth cell and predicted cell is calculated as the ratio of the total length of the longest common string sequences to the length of the ground-truth cell content string. The similarity of the aligned ground truth and predicted table substructures is estimated by the intersection-over-union (IOU) of the aligned bounding boxes.

1 $$\begin{aligned} \text {GriTS}_f(\textbf{A}, \textbf{B}) = \frac{2 \cdot \sum _{i,j} f(\tilde{\textbf{A}}_{i,j}, \tilde{\textbf{B}}_{i,j})}{|\textbf{A}| + |\textbf{B}|}, \end{aligned}$$

2 $$\begin{aligned} \text {GriTS-P}_f(\textbf{A}, \textbf{B}) = \frac{\sum _{i,j} f(\tilde{\textbf{A}}_{i,j}, \tilde{\textbf{B}}_{i,j} )}{|\textbf{B}|} \end{aligned}$$

3 $$\begin{aligned} \text {GriTS-R}_f(\textbf{A}, \textbf{B}) = \frac{\sum _{i,j} f(\tilde{\textbf{A}}_{i,j}, \tilde{\textbf{B}}_{i,j} )}{|\textbf{A}|} \end{aligned}$$

## Results

To evaluate the table detection performance via the Table Transformer TD model, 1000 randomly selected preprints (between 2019 and 2023) are further filtered to 143 PDF documents (paper body and supplementary files) containing the RRID prefix. After conversion to an image, the Table Transformer TD model is applied to each page of the 143 PDF documents resulting in 2626 table candidates for which the bounding box content is saved as a separate image for curation. A single annotator looked through each table candidate image and decided whether the bounding box contents were an actual table. Of the 2626 table predictions, only 356 were actual tables with an accuracy of 13.6%. Most errors seem to fall into one of these categories: 1) pages with line numbers (a common occurrence in preprints), 2) numbered references, 3) figure captions, 4) first-page author lists, and 5) other kinds of lists. However, if a page contains any table, the detection rate is much higher as reported in [14]. This result demonstrates the need for the detection of key resource table-containing pages before applying the Table Transformer TD model.

### Key Resource Table Page Candidate Detection Performance

The test performances of the key resource table candidate page detection systems are summarized in Table 1. BoW feature only SVM achieves very high precision but lower recall rate. The two-level stacked generalizer model performs slightly better by increasing recall at the expense of some precision. To evaluate the model in a larger setting, 200 new randomly selected preprints were passed through the stacked generalizer model and results were curated by student curators. Compared to the smaller test case, only a small drop in precision and recall is observed, indicative of the robustness of the page detection classifier.

**Table 1:** Test performance of key resource table page candidate detection systems

Model	Precision	Recall	$F_1$
BoW only SVM	100%	67%	80%
Stacked Generalizer model	92%	73%	81%
Stacked Generalizer model over larger test set	84%	67%	75%

### Key Resource Table Extraction Performance

The table content and topology extraction performance of the four table extraction pipelines introduced together with the GROBID baseline on the 100 gold standard key resource tables are summarized in Table 2. All pipelines introduced outperformed the GROBID baseline by a significant margin. GROBID could only detect 19 of the 100 gold standard tables, resulting in low GriTS table similarity scores. The best-performing pipeline, RTE-LM with Table LM-based row merging, significantly outperformed the OCR pipeline (RTE-OCR) using the two-tailed t-test with a p-value of 0.01. Of the introduced pipelines, the pipeline that uses both column and row bound information from the Table Transformer TSR model (RTE-Row) was the worst performing model. This is mainly due to the distances between the rows being usually much smaller than the column distances, resulting in row boundary errors and cropped bounding boxes leading to PDF text to table cell alignment issues. Due to the row over-segmentation, the column information-only RTE-Col pipeline showed lower performance than the RTE-OCR pipeline, especially for content and topology precision, namely $\text {GriTS-P}_{cont}$ and $\text {GriTS-P}_{top}$. RTE-LM pipeline with character-level Table LM for row merging remedied the row over-segmentation issue of the RTE-Col pipeline relying on the learned syntactic and semantic properties of the language of scientific tables.

**Table 2:** Test performance of key resource content extraction pipelines

Pipeline	$\text {GriTS}_{cont}$	$\text {GriTS-P}_{cont}$	$\text {GriTS-R}_{cont}$	$\text {GriTS}_{top}$	$\text {GriTS-P}_{top}$	$\text {GriTS-R}_{top}$
Grobid	0.1162	0.1190	0.1380	0.1341	0.1381	0.1643
RTE-Col	0.7450	0.6804	0.8907	0.7842	0.7103	0.9565
RTE-Row	0.5893	0.7879	0.6196	0.6753	0.9091	0.6196
RTE-OCR	0.7553	0.7483	0.7798	0.8496	0.8419	0.8805
RTE-LM	**0.8981**	**0.8898**	**0.9172**	**0.9262**	**0.9158**	**0.9504**

$\text {GriTS}_{f}$, $\text {GriTS-P}_{f}$ and $\text {GriTS-R}_{f}$ are defined in Eqs. 1, 2 and 3, respectively. Pipelines include Grobid only, RTE-Col (using only TSR column location information), RTE-Row (using both TSR column and row location information), RTE-OCR (fully image based using OCR for cell contents), RTE-LM (using only TSR column information together with character level table language model based row merging)

## Discussion

Although RTE-OCR was the second best performing pipeline, OCR usually results in point errors within recognized words, which could make them not recognizable. This is especially a problem for key resources that are required to be identifiable for the reproducibility of a study. For example, the nucleotide sequence ’GCACTTCATCCTTTGG G’ recognized by the OCR misses a portion of the full sequence ’GCACTTCATCCTTTGGTTTTG.’ OCR can mangle a key resource name, e.g., ’Alexa antl-Maddil IQ’ instead of ’Alexa anti-Rabbit IgG’. OCR errors are most detrimental if they occur in a key resource identifier such as a catalog number, e.g., ’alt 1/404/6’ instead of ’Cat# 1745478’. However, the RTE-LM pipeline extracts the table cell contents from the text content of the PDF document, always resulting in correct text in the detected table cells.

Out of the four resource table extraction pipelines introduced, we recommend the best performing RTE-LM for general use for articles in PDF format that are not generated by scanning the articles. This is generally the case for almost all articles after the 1990s. For scanned papers in PDF format, the RTE-OCR pipeline is the best choice.

The introduced key resource extraction pipelines are implemented in combination of Java and Python languages and are publicly available. For practical usage, the system is developed in a client/server fashion as a web service. On average, a preprint paper can be processed in under a minute without using a GPU, which if available will further decrease the processing time. The system was recently deployed on cloud for production use as part of our key resource detection and identification system for BioRxiv. In this setting, the system has processed more than 15,000 preprints submitted to BioRxiv, and these results will be made available to preprint authors starting in 2025.

We acknowledge that several key limitations of our current study are present. Due to the time-consuming nature of the annotation task, the gold set is relatively small and thus may not be representative of all types of resource tables. Additionally, the table extraction pipelines cannot be directly used for other kind of tables, such as statistical tables. To adapt the pipelines to different types of tables, the table page detection classifier needs to be retrained with annotated pages containing the relevant tables. Also, the row merger classifier needs to be fine-tuned with synthetic training data generated from a pertinent type of table content.

We also acknowledge that the resource table detection classifier training/test set is relatively small. We have validated its generalization ability using a second larger test set of 200 articles, as shown in Table 1. We plan to extend this annotated dataset when more annotation resources are available.

In the future, we are planning to extend the system also to handle column over-segmentation by learning to merge consequent column contents, if necessary. Column over-segmentation is usually a lesser issue than row over-segmentation (as observed during the gold set annotation) since it is only observed with table section headers, which occur much less frequently than the column overflows, resulting in row over-segmentation.

Another future direction is to fine-tune our table-specific language model for post-OCR error correction [32] for tables only available as scanned documents for older papers where only the image modality is available to exploit.

## Conclusion

While tables are a key visual representation of text and numerical data that are highly useful for human beings because they provide immediate feedback to authors about missing information, tables are not easy to extract by conventional approaches. The fact that tables generally contain non-duplicate information in a format that is not English, per se, meant that optical character recognition approaches were insufficient because any error of a character could not be corrected based on context of the character. If, for example, a “g” becomes an “a” in an English word, the word can be directed as being misspelled, but no such misspelling detection is possible when the same error is made in a DNA sequence, a catalog number or in an organism name where symbols superscripts and subscripts are very common, for example this fly name “Pry[+t7.2]=hsFLP12, y[1] w[*]; Pw[+mW.hs]=GawBap[md544] Pw[+mW.hs]=GawBptc[559.1]/CyO, Pw[+mC]=ActGFPJMR1; Pw[+mC]=UAS-TagBFP9D” contains the fly genomic nomenclature in which nearly any character may be substituted for any other.

We introduced four pipelines for key resource table extraction from biomedical documents in PDF format. Our approach reconstructs key resource tables using image-level table detection and structure detection generated table boundary, column (and row) bounding box information together with PDF text alignment. To remedy row over-segmentation resulting from overflowing table cell contents, we introduced a language modeling (LM) based row merging solution where a character-level GPT model is pre-trained on more than 11 million scientific table contents from PMC OAS. All introduced pipelines significantly outperformed the GROBID baseline, while our Table-LM based row merging pipeline, significantly outperformed all other pipelines including our OCR-based pipeline.

## Data Availability

The annotated data (including gold set used for evaluations) can be found at https://github.com/SciCrunch/key resource table extractor. The Table LM model and row merger classifier model can be found at Zenodo (https://doi.org/10.5281/zenodo.13924310). The GLOVE word/phrase embeddings used for key resource page detection classifier are available from https://doi.org/10.5281/zenodo.13924223.

## Code availability

The key resource extraction pipeline code together with all the annotated data (including gold set used for evaluations) can be found at https://github.com/SciCrunch/key_resource_table_extractor. The Table LM pre-training and row merger fine-tuned classifier code is available at https://github.com/SciCrunch/table_lm.
